# Rho kinase activity controls directional cell movements during primitive streak formation in the rabbit embryo

**DOI:** 10.1242/dev.111583

**Published:** 2015-01-01

**Authors:** Viktoria Stankova, Nikoloz Tsikolia, Christoph Viebahn

**Affiliations:** Institute of Anatomy and Embryology, University Medical Centre, Georg August University of Göttingen, Göttingen 37079, Germany

**Keywords:** Amniote evolution, Cell migration, Mammalian gastrulation, Mesoderm formation, Planar cell polarity, Primitive streak

## Abstract

During animal gastrulation, the specification of the embryonic axes is accompanied by epithelio-mesenchymal transition (EMT), the first major change in cell shape after fertilization. EMT takes place in disparate topographical arrangements, such as the circular blastopore of amphibians, the straight primitive streak of birds and mammals or in intermediate gastrulation forms of other amniotes such as reptiles. Planar cell movements are prime candidates to arrange specific modes of gastrulation but there is no consensus view on their role in different vertebrate classes. Here, we test the impact of interfering with Rho kinase-mediated cell movements on gastrulation topography in blastocysts of the rabbit, which has a flat embryonic disc typical for most mammals. Time-lapse video microscopy, electron microscopy, gene expression and morphometric analyses of the effect of inhibiting ROCK activity showed – besides normal specification of the organizer region – a dose-dependent disruption of primitive streak formation; this disruption resulted in circular, arc-shaped or intermediate forms, reminiscent of those found in amphibians, fishes and reptiles. Our results reveal a crucial role of ROCK-controlled directional cell movements during rabbit primitive streak formation and highlight the possibility that temporal and spatial modulation of cell movements were instrumental for the evolution of gastrulation forms.

## Abstract (precis)

**Summary**: ROCK regulates cell motility, polarisation, intercalation and division in gastrulating rabbit embryos, revealing a role for Wnt-PCP signalling in primitive streak formation.

## INTRODUCTION

Typical landmarks of gastrulation are the circular blastopore in amphibians and the straight primitive streak in amniotes (birds and mammals). Both mark the initial site of mesoderm formation by epithelial-mesenchymal transition (EMT) and, thereby, create the ‘milieu intérieur’ (Bernard, 1859) as the basis for all internal organ anlagen. Despite their disparate forms, these landmarks have long been regarded as homologous structures (Kollmann, 1886; Pasteels, 1940; Stern, 2004). Recent analysis of reptile gastrulation (Coolen et al., 2008; Bertocchini et al., 2013) revealed an intermediate gastrulation form consisting of a blastopore in addition to a blastoporal plate, a broad posterior structure considered to be homologous to the primitive streak (Gilland and Burke, 2004).

Among the best-studied cellular mechanisms for determining early embryonic form are: (1) convergent extension (CE) of the axial mesoderm in *Xenopus* (Keller and Danilchik, 1988), (2) ‘polonaise’ cell movements (Wetzel, 1929) and (3) medio-lateral cell intercalation (Voiculescu et al., 2007) in the epiblast prior to primitive streak formation in the chick. The latter two types of cell movement also occur in the mammotypic flat embryonic disc of the rabbit (*Oryctolagus cuniculus*), and were specified as L- and U-turn movements and as extended cell intercalation (also known as processional cell movement), respectively (Halacheva et al., 2011); nevertheless, the primitive streak of the mouse seems to form via local PCP-independent EMT in the epiblast (Williams et al., 2012). Both medio-lateral cell intercalation and CE movements are dependent on the Wnt-planar cell polarity (Wnt-PCP) pathway, which thus controls anterior-posterior axis elongation during both primitive streak formation and notochord formation (Heisenberg et al., 2000; Wallingford et al., 2002a; Voiculescu et al., 2007; Mahaffey et al., 2013).

The early activation of PCP-dependent cell intercalation towards the midline in the posterior epiblast of the chick is thought to be a cellular mechanism for the evolutionary emergence of the primitive streak in amniotes (Voiculescu et al., 2007). However, birds and mammals start gastrulation under different morphological preconditions: an anterior thickening called ‘anterior pregastrular differentiation’ in mammals (Hassoun et al., 2009) versus a posterior thickening (Koller's sickle) in birds (Stern, 2004). An intriguing question is thus, whether cellular rearrangements controlled by the PCP pathway might play a role in the emergence of the primitive streak in mammals as well. In the present study, we examined the role of pre-gastrulation cell movements in the rabbit embryonic disc during primitive streak formation by chemical inhibition of the Rho kinase (ROCK) (Uehata et al., 1997); ROCK is a downstream effector in the Wnt-PCP pathway influencing directional cell movement by remodeling the actomyosin cytoskeleton (Habas et al., 2001; Winter et al., 2001).

## RESULTS AND DISCUSSION

### Planar cell movements shape the mammalian primitive streak

**Fig. 1.**
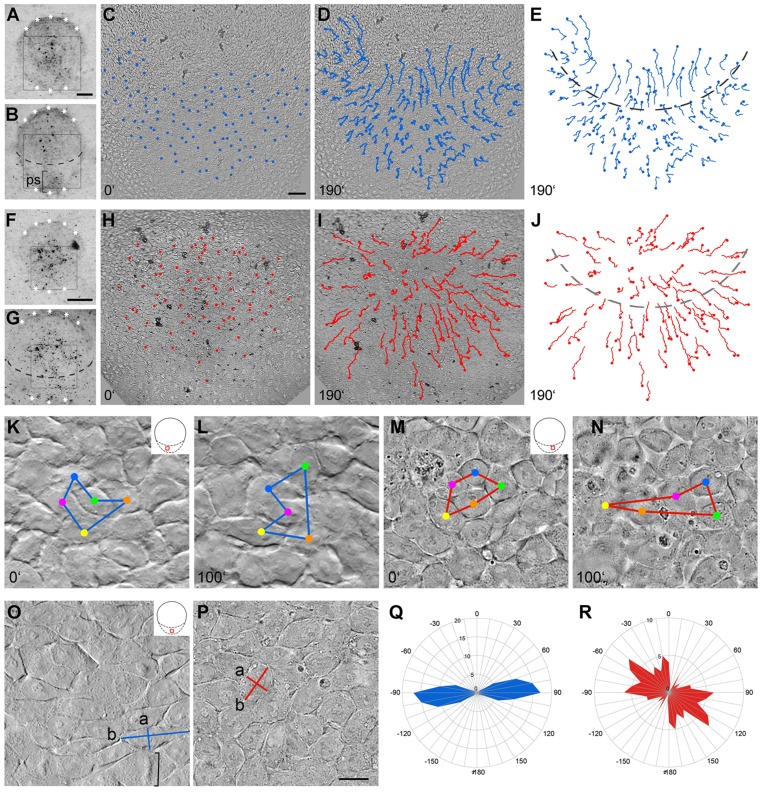
**Pre-gastrulation cell movements are impaired by ROCK inhibition.** (A-D,F-I) Dorsal views of control (A-D) and 40 µM ROCK-inhibited (F-I) rabbit embryonic discs taken at the beginning (A,C,F,H) and the end (B,D,G,I) of the time-lapse movie (supplementary material Movies 1,2). Asterisks mark embryonic disc borders. (E,J) Paths of the traced cells relative to the posterior gastrula extension (PGE) area (anterior border marked by dashed lines). (K,L) Processional cell movement in control embryo (see the cell marked in red passing between the cells marked in blue and yellow). (M,N) Cell intercalation in treated embryo (see the cell marked in red squeezed between the cells marked in green and orange in M,N). (O,P) High magnification of DIC recordings, showing cell form and protrusions defined by the quotient of the long cell axis (b) divided by the short cell axis (a) in control (O) and treated (P) embryo. Square bracket in O marks metaphase plate. (Q,R) Quantitative analysis of the orientation of the longest cell protrusions related to the anterior-posterior (AP) axis (0°±180°) in control (Q) and in treated (R) embryos using measurements of the angles between the longest cell axis and the AP axis. Radial graduation shows the number of cells that fall within a specific angular region and orbital graduation shows the angles. Scale bars: 250 µm in A for A,B; 50 µm in C for C-E,H-J; 175 µm in F for F,G; 5 µm in P for K-P.

Using time-lapse microscopy of pre-gastrulating rabbit blastocysts, inverted L- and U-turn movements of cells towards the posterior midline of the embryonic disc were revealed (1) to complement the L- and U-turn movements described previously (Halacheva et al., 2011), and (2) to contribute to the elongation of the primitive streak (Fig. 1A-E; supplementary material Movie 1). This is in contrast to the mouse, in which the rare appearance of L- and U-turns (Williams et al., 2012) might be accounted for by the restricted space in the rodent egg cylinder.

To define how the complex planar cell movements observed in the rabbit embryonic disc might contribute to the formation of the mammalian primitive streak, we used the chemical compound Y-27632 (Uehata et al., 1997) to interfere with the activity of Rho kinase (ROCK), which is expressed in the epiblast at the incipient gastrulation stages (supplementary material Fig. S2). This treatment specifically affected lateral-to-medial cell movements in the posterior gastrula extension (PGE), the prospective primitive streak area of the rabbit embryonic disc (Fig. 1F-J; supplementary material Movie 2): extended centrifugal cell movements within the PGE prevailed and led to concentric widening of the PGE area, whereas the midline cells anterior to the PGE border (see dashed lines in Fig. 1E,J) showed short tracks similar to the L- and U-turns observed during normal development (Halacheva et al., 2011). Intercalation of neighbouring cells in the PGE area led to cellular spreading along the medio-lateral (ML) axis (Fig. 1M,N), whereas in control embryos cell intercalation contributed to elongation of the anterior-posterior (AP) axis (Fig. 1K,L). Processional cell movements (Halacheva et al., 2011) seen in control embryos (Fig. 1K,L) were not observed in treated embryos (Fig. 1M,N). With regard to the shape of individual cells, epiblast cells in the PGE of control embryos showed planar elongation (Fig. 1O) oriented specifically along the ML axis (Fig. 1Q), whereas epiblast cells from the same region in treated embryos showed reduced planar elongation (Fig. 1P) and no preferred orientation of the long cell axis (Fig. 1R; *P*<0.0001 by independent *t*-test with unequal variances). Finally, these differences in cell behaviour were mirrored by regular cortical actin distribution in control embryos (supplementary material Fig. S1D) and abnormal actin foci in the cell periphery in treated embryos (supplementary material Fig. S1E).

Orientation and frequency of cell division were also altered in embryos treated with a ROCK inhibitor: the number of dividing cells in a given area of the PGE was approximately halved (supplementary material Fig. S1A) compared with that of the normal counterparts (compare with Halacheva et al., 2011), and metaphase plates lost their orientation parallel to the AP axis (Halacheva et al., 2011), i.e. the angles of the metaphase plates with the AP axis varied broadly in treated embryos (supplementary material Fig. S1B,C). In summary, impaired planar cell behaviour consisting of extended cell intercalation, cell polarisation and polarized cell division resulted in an abnormally broad PGE area of treated embryos, thus emphasizing the role of directional cell movement in the development of the rabbit primitive streak.

### Modified primitive streak combined with regular organizer specification

**Fig. 2.**
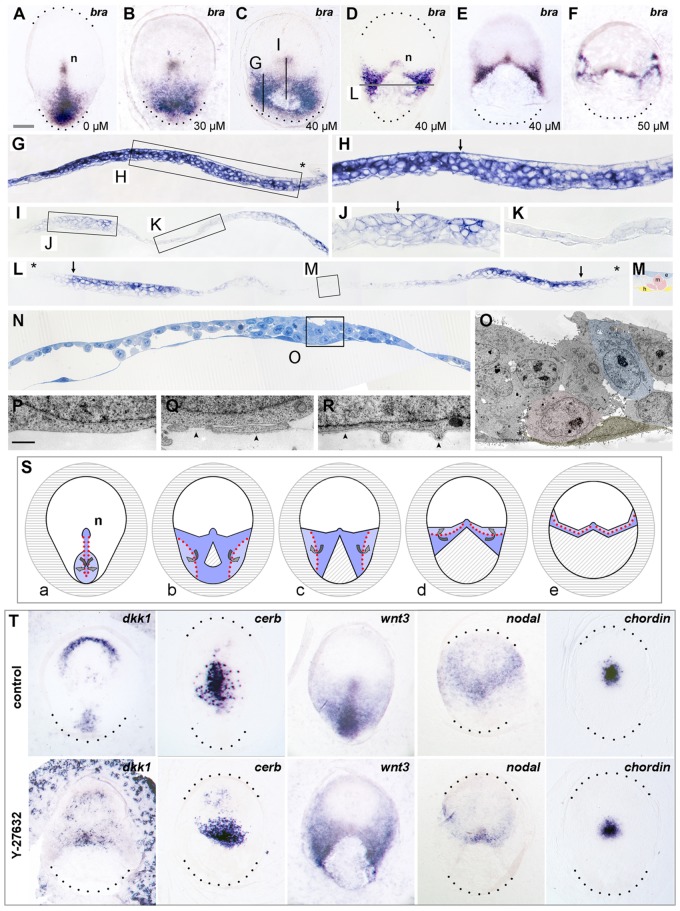
**Dose-dependent reshaping of primitive streak.** (A-F,S,T) Dorsal views of control (A, Sa and top row of T) and ROCK-inhibited embryos (B-F, Sb-Se and bottom row of T) analysed for *brachyury* (A-F, Sa-Se), *wnt3*, *nodal*, *dickkopf1*, *cerberus* and *chordin* (all in T) in sagittal (G-K) or transversal (L-R) sections. Anterior is to the top in dorsal views and to the left in sagittal sections. Black dots mark posterior embryonic disc borders. Asterisks mark epiblast-trophoblast border. Arrows mark the position of the epithelio-mesenchymal hinge (EMH) in H and L and the chordoneural hinge in J. (M) High magnification of occasional mesoderm cells (red) in the *brachyury*-negative area shown in L. (N) Transversal semithin section from presumptive primitive streak area showing one half of a treated embryo (midline is near the right edge). Box indicates the area shown in O. (O) Ultrathin section showing bottle cell (blue), mesodermal cell (pink) and hypoblast cell (yellow). (P,Q) Epiblast cells from the PGE area (P) and from the anterior half of the embryonic disc (Q). (R) Trophoblast cell. Arrowheads point to the existing basement membrane (Q,R). (S) Drawing of mesoderm displacement by ROCK inhibition: Mammotypic (a), reptilian-like (b), amniote precursor-like (c,d) and amphibian-like or teleost-like (e) gastrulation centres. Dark blue colour marks EMT area. Red dotted lines indicate the border of the EMH. Light blue colour marks the EMT-free epiblast. Curved arrows indicate the dorso-ventral direction of EMT and subsequent lateral mesoderm migration. e, epiblast; h, hypoblast; m, mesoderm; n, node. Scale bar in A: 100 µm for A-F, T; 50 µm for G, I, L; 25 µm for H, J, K, M; scale bar in P: 10 µm for N, 2 µm for O, 1 µm for P-R.

Intriguingly, the ROCK inhibition caused deformation of the rabbit primitive streak in a dose-dependent manner. Expression of *brachyury* (Fig. 2A-F), a gene controlling mesoderm formation in vertebrates (Herrmann et al., 1990), revealed that low doses of the ROCK inhibitor produced a *brachyury-*expression pattern (Fig. 2B) similar to that seen in the chick after interference with the PCP-component dishevelled (Voiculescu et al., 2007). At higher inhibitor concentrations, circular ‘blastopore-like’ *brachyury*-negative areas of different diameters appeared in the centre of the primitive streak-forming area (Fig. 2C-E; see also supplementary material Table S1), and the shape of these abnormal primitive streaks could be classified into three groups (see supplementary material Table S2). Treatment with higher inhibitor concentrations resulted in mesoderm-forming areas in which the two flanking, *brachyury*-expressing domains formed an arc-like equatorial shape connected to the node (Fig. 2F). The respective anterior and posterior primitive streak markers *cerberus1* (*Cer1*) and *dickkopf1* (*Dkk1*; compare with Idkowiak et al., 2004) showed widened expression domains extending towards the primitive node, whereas their anterior expression domains in the hypoblast remained unaffected (Fig. 2T). The expression patterns of the genes coding for the mesoderm-inducing signals Wnt3 and nodal (compare with Morkel et al., 2003) showed posterior widening and anterior expansion (Fig. 2T) when compared with control embryos. In view of the rapid response to ROCK inhibition, these modified expression patterns of genes involved in mesendoderm formation are probably the result of cellular translocation rather than of a change in cell identity. Superficially, these abnormal expression patterns mirror different vertebrate gastrulation forms: the reptilian blastopore and blastoporal plate (Coolen et al., 2008; Bertocchini et al., 2013; compare with Fig. 2C,Sb), the amphibian blastopore (Gont et al., 1993), the teleost germ ring (Martin and Kimelman, 2008; compare with Fig. 2F,Se) and putative transient gastrulation forms of hypothetical amniote precursors (Arendt and Nübler-Jung, 1999; Fig. 2D,E,Sc,Sd).

**Fig. 3.**
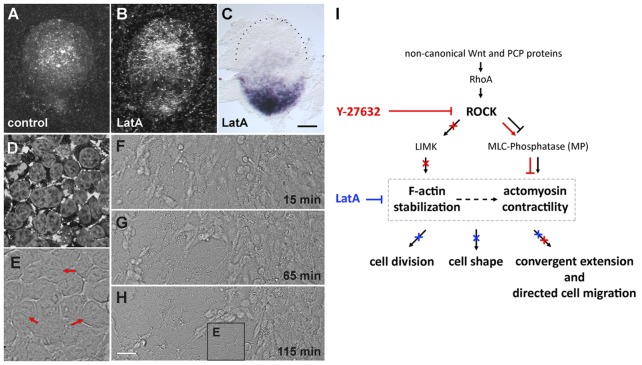
**Latrunculin A affects actin cytoskeleton in the whole embryonic disc.** (A-C) Dorsal views of control (A) and LatA-treated (B,C) embryos. (C) *Brachyury* expression. (D) Phalloidin-TRITC/DAPI staining of LatA-treated embryo. (E-H) DIC frames of a time-lapse movie showing both epiblast and ablation of hypoblast in the PGE area of LatA-treated embryo (supplementary material Movie 3). Red arrows in E indicate unseparated daughter cells. (I) Schematic of the effects of ROCK-inhibitor (Y-27632) and LatA on cellular level. Scale bar in C: 250 µm for A-C and 10 µm for D, E; scale bar in H: 50 µm for F-H.

At the histological level, the longitudinal band of tissues displaying EMT in the primitive streak (Viebahn et al., 1995) was replaced by a thin, basically two-layered *brachyury-*negative region (Fig. 2K,L) in all experimental embryonic discs (Fig. 2G-R). In support of the fact that both EMT and the preceding breakdown of the epiblast basement membrane are dependent on ROCK activity (Nakaya et al., 2008; Marinari et al., 2012), transmission electron microscopy of treated embryos revealed: (1) complete absence of basement membrane in the PGE (compare with Fig. 2P-R), (2) ectopic areas of EMT lying lateral to the midline (Fig. 2N,O) and (3) an ‘epithelio-mesenchymal hinge’ (EMH) as the lateral border of EMT, correlating with the transition of *brachyury-*positive to *brachyury-*negative epiblast (compare with arrows in Fig. 2H,L and dotted lines in Fig. 2S). EMT thus occurred independently of primitive streak shape (Stern and Canning, 1990; Stern et al., 1995; Alev et al., 2013).

Dorso-ventral patterning in the experimental rabbit blastocysts remained undisturbed: the organizer region (Fig. 2T) expressed the notochord marker *chordin* (Sasai et al., 1994) and displayed the normal close apposition of the prospective neuroectoderm and the epithelialized notochordal process (compare with Fig. 2J) anterior to the chordoneural hinge (Cambray and Wilson, 2002). Regular *brachyury* and *chordin* expression and notochord formation in the node area of ROCK-inhibited embryos also highlight the *brachyury*-negative anterior half of the normal primitive streak (Hue et al., 2001; Viebahn et al., 2002), where EMT appears to occur independently of *brachyury* expression. In summary, ROCK-inhibited embryos displayed a dose-dependent widening of the primitive streak with ectopic mesoderm formation but proper organizer and notochord specification. This deformation of the primitive streak might be caused by altered lateral-to-medial cell movements and cell intercalation. However, whether the graded effect of ROCK inhibitor is mediated by individual cells or groups of neighbouring cells remains to be clarified.

### ROCK inhibition specifically acts on mammalian primitive streak formation

To test whether the transformation of the primitive streak in ROCK-inhibited embryos was caused by overall suppression of cytoskeleton-dependent processes, actin polymerisation was inhibited by latrunculin A (LatA; Spector et al., 1989). This led to abnormal intracellular focal actin accumulation (Fig. 3D), incomplete cytokinesis (Fig. 3D,E) and impairment of epiblast cell movements throughout the whole embryonic disc (Fig. 3F-H; supplementary material Movie 3). Additionally, LatA-treated embryos displayed neither *brachyury*-negative areas nor posterior widening, as seen after ROCK-inhibition (compare Fig. 3C and Fig. 2B-E). This finding and the general suppression of cellular activities were in marked contrast to the specific effects of ROCK-inhibition, in which oriented cell intercalations were lost in the posterior half of the embryonic disc. These results provide indirect support for the involvement of planar cell polarity in primitive streak formation of the rabbit and are in line with the view that PCP-dependent cell movements contribute to elongation of the AP axis in the chick and frog (Voiculescu et al., 2007; Wallingford et al., 2002b). However, further experiments, including genetic knockdown of individual PCP components and transcriptome analysis of widened primitive streaks, might help to clarify molecular mechanisms of primitive streak formation and functional attributes of ectopic EMT domains.

### Subtle changes in cell rearrangement and the evolution of gastrulation topography

**Fig. 4.**
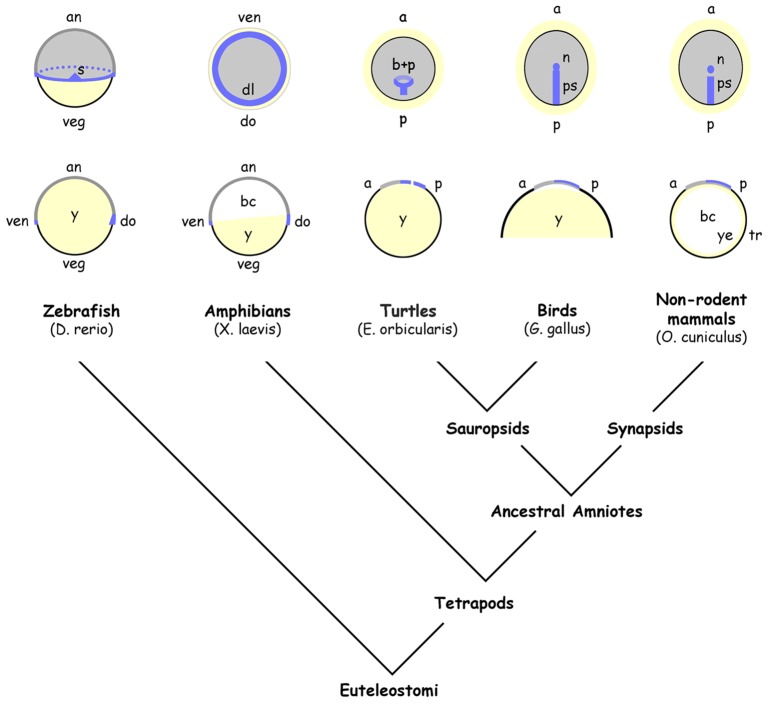
**Gastrulation forms in vertebrates.** Dorsal (animal for *Xenopus*) and sagittal views of zebrafish at 50% epiboly (Kimmel et al., 1995), *Xenopus* at stage 10 (Nieuwkoop and Faber, 1994), turtle at stage 0a (Coolen et al., 2008), chick at early stage 4 (Tsikolia et al., 2012) and rabbit at early stage 4 (Viebahn, 2004). a, anterior; an, animal; bc, blastocoel; b+p, blastopore and blastoporal plate; dl, dorsal blastopore lip; do, dorsal; n, node; p, posterior; ps, primitive streak; s, embryonic shield; tr, trophoblast; veg, vegetal; ven, ventral; y, yolk; ye, yolk sac epithelium. Blue marks *brachyury* expression and yellow marks the yolk or the yolk sac epithelium.

Evolutionary transformation of the circular blastopore into the straight primitive streak might have occurred through displacement of the mesoderm-forming domain to the prospective posterior pole (compare with Fig. 4) under the ‘pressure’ of an increasing yolk mass (Arendt and Nübler-Jung, 1999). Here, we propose that stepwise morphogenetic changes associated with different primitive streak shapes (Fig. 2B-F) could explain the evolutionary invention of the straight primitive streak starting with the circular ancestral blastopore. This concept includes: (1) absent lateral-to-medial planar cell movements associated with the extreme form of a widened gastrulation centre (Fig. 2D,E,Sc,Sd) and resembling the situation in a hypothetical amniote precursor (Arendt and Nübler-Jung, 1999); (2) moderate lateral-to-medial planar cell movements producing the intermediate form of a broad primitive streak (Fig. 2C) and leading to a reptilian-style gastrulation topography (Arendt and Nübler-Jung, 1999; Bertocchini et al., 2013); and (3) a full complement of cell rearrangements in conjunction with oriented cell division, leading to cellular convergence towards the midline and elongation of the amniote primitive streak. An important part of this concept is the spatio-temporal shift of PCP-dependent cell rearrangements to the pre-gastrulation stage (Voiculescu et al., 2007) and to the posterior area of the amniote embryonic disc, a conjecture we set out to examine in the present study.

Because reptiles are descendants of ancestral amniotes and are considered to be basal to birds (Fig. 4), gastrulation of the enigmatic common ancestor of birds and mammals would most likely be accomplished by a blastoporal plate instead of a primitive streak. The primitive streak could thus have emerged twice in amniotes (Gilland and Burke, 2004) as a result of a morphogenetic constraint (Richardson and Chipman, 2003). The apparent kinship of experimentally generated rabbit primitive streaks with the reptilian blastoporal plate, and the rise of reptilian model organisms (Coolen et al., 2008; Bertocchini et al., 2013), now call for further analysis of the PCP pathway in reptiles to determine whether cellular motility might have enabled convergent evolution of vertebrate gastrulation while leaving the specification of the organizer unaffected.

## MATERIALS AND METHODS

### Embryo culture

Young adult New Zealand White rabbits (Charles River Laboratories) (2.5 kg body weight) were given 0.8 µg of buserelin, a gonadotropin-releasing hormone analogon (Receptal; Intervet) intramuscularly (i.m.) at least four weeks prior to mating. To increase ovulation and fertility rate, a second dose of buserelin (0.8 µg) was given immediately after mating. At 6.2 days post coitum (dpc), uteri were removed by Caesarean section after intravenous administration of an overdose (320 mg) of pentobarbital sodium (Narcoren; Merial) and immersed in warm (37°C) phosphate-buffered saline (PBS). Blastocysts were flushed from uteri using warm PBS, transferred to warm Ham's F10 culture medium (Biochrom) supplemented with 20% fetal calf serum, 50 IU penicillin and 50 mg/ml streptomycin (both Biochrom), and cultured individually in 2 ml of medium. For interfering with cell movements, the following substances were added: (1) Y-27632 dihydrochloride (R&D Systems) in PBS for inhibition of ROCK; and (2) latrunculin A (LatA; Sigma-Aldrich) in dimethyl sulfoxide (DMSO; Sigma-Aldrich) for inhibition of actin polymerisation at concentrations of 30, 40 and 50 µmol from 4 mmol/l Y-27632 stock and of 0.5, 1 and 2.5 µmol from 2.5 mmol/l LatA stock, respectively. Control embryos were cultivated in medium with respective carrier volumes of PBS or DMSO. All cultured embryos were incubated at 37°C under 5% CO_2_ for 18 h (Y-27632) or 6 h (LatA) to reach early gastrulation stages. Embryo cultures were stopped by fixation with 4% paraformaldehyde (PFA) in PBS for 1 h at room temperature.

### *In situ* hybridisation probes

PCR products of rabbit cDNA corresponding to the correct size of *brachyury*, *dickkopf1* and *cerberus1* (Idkowiak et al., 2004), as well as *Wnt3* (GenBank accession number DQ786778.1), *nodal* (Fischer et al., 2002) and *chordin* (GenBank accession number: AY575210.1) mRNA were cloned and sequenced using standard conditions (compare with Idkowiak et al., 2004). Degenerated primer combinations for *chordin* (710 bp) were 5′-CATGGTGTGGTRAARGAYYTNGARC-3′ (forward) and 5′-ACA-CGSACNGGYTGNGCRC-3′ (reverse); and for Wnt3 (372 bp) were 5′-GGGGAATTCCARGARTGYAARTGYCAT-3′ (forward) and 5′-AAA-ATCTAGARCARCACCARTGRAA-3′ (reverse). For *in situ* hybridisation of the gene coding for Rho-associated coiled-coil containing protein kinase 1 (ROCK1), a synthetically produced cRNA probe corresponding to bp 3781-4363 of rabbit *ROCK1* (GenBank accession number: NM_001082367.1) was obtained from GeneCust (Dudelange, Luxemburg).

### Imaging and histology

Using differential-interference contrast (DIC) microscopy, cell movements of embryos treated with Y-27632 or LatA were recorded with an inverse Axiovert 200M microscope equipped with an incubation chamber, an Axiocam MRm camera and an AxioVison software (all from Zeiss) taking one frame every one minute for up to 4 h (compare with Halacheva et al., 2011). Cell movements of untreated embryos were recorded using the same conditions. Image analysis of the time-lapse series of seven treated embryos and at least seven controls was carried out using ImageJ software (NIH). *In situ* hybridisation of whole-mount rabbit embryos was carried out following standard protocols (Idkowiak et al., 2004). After *in situ* hybridisation, transverse and sagittal sections (5 µm) were cut from embryonic discs embedded in Technovit medium (Heraeus-Kulzer). For light and electron microscopy, embryos treated with the ROCK inhibitor were fixed in 1.5% PFA and 1.5% glutaraldehyde (GA) in PBS, post-fixed in 1% osmium oxide (OsO_4_) in PBS and subsequently embedded in araldite (Hassoun et al., 2009). Serial semithin sections (1 µm) of the araldite-embedded embryonic discs were cut in the transverse plane. For transmission electron microscopy, selected semithin sections were re-embedded in araldite (Viebahn et al., 1995), sectioned at 70 nm, contrasted with uranyl acetate and lead citrate and analysed with a TEM 900 transmission electron microscope (Zeiss). For visualizing the actin cytoskeleton, treated embryos were stained with phalloidin-TRITC (Sigma-Aldrich) in PBS diluted to 1:500 and counterstained with DAPI in PBS (Serva Electrophoresis) diluted to 1:5000.

### Quantitative analysis of cell movement, cell shape and cell division

To evaluate cellular elongation and planar polarisation, the longest and the shortest cell axes (see Fig. 1O, b; and Fig. 1O, a, respectively) were measured in still pictures of time-lapse movies in control embryos (*n*=3) and embryos treated with ROCK-inhibitor (*n*=3). The quotient of the former (‘b’) divided by the latter (‘a’) was calculated in 67 cells from control embryos and in 61 cells from treated embryos to obtain mean quotients and standard deviations. To analyse the orientation of elongated cells and of the cell divisions in control and treated embryos, the angles of the longest cell axes and of the metaphase plates with respect to the AP axis were measured and grouped in intervals of 10°. Each group of angles (α) was depicted with its opposite group of angles (α+180°) in rose diagrams using Excel software (Microsoft).

## Supplementary Material

Supplementary Material
