# Supplementary material for: Rho kinase activity controls directional cell movements during primitive streak formation in the rabbit embryo
Source: Development. 2015 Jan 1;142(1):92–8. doi: 10.1242/dev.111583 (PMC4299133; doi:10.1242/dev.111583)
Supplement: Supplementary Material [file supp_142_1_92__index.html]

Supplementary Material 

# Rho kinase activity controls directional cell movements during primitive streak formation in the rabbit embryo

## DEV111583 Supplementary Material

**Files in this Data Supplement:**

- Supplementary Material
