# Supplementary material for: Rho kinase activity controls directional cell movements during primitive streak formation in the rabbit embryo
Source: Development. 2015 Jan 1;142(1):92–8. doi: 10.1242/dev.111583 (PMC4299133; doi:10.1242/dev.111583)
Supplement: Supplementary Material [file supp_142.1.92_DEV111583supp.pdf]

DEV111583 Supplementary Material

Legends of Supplementary Figures

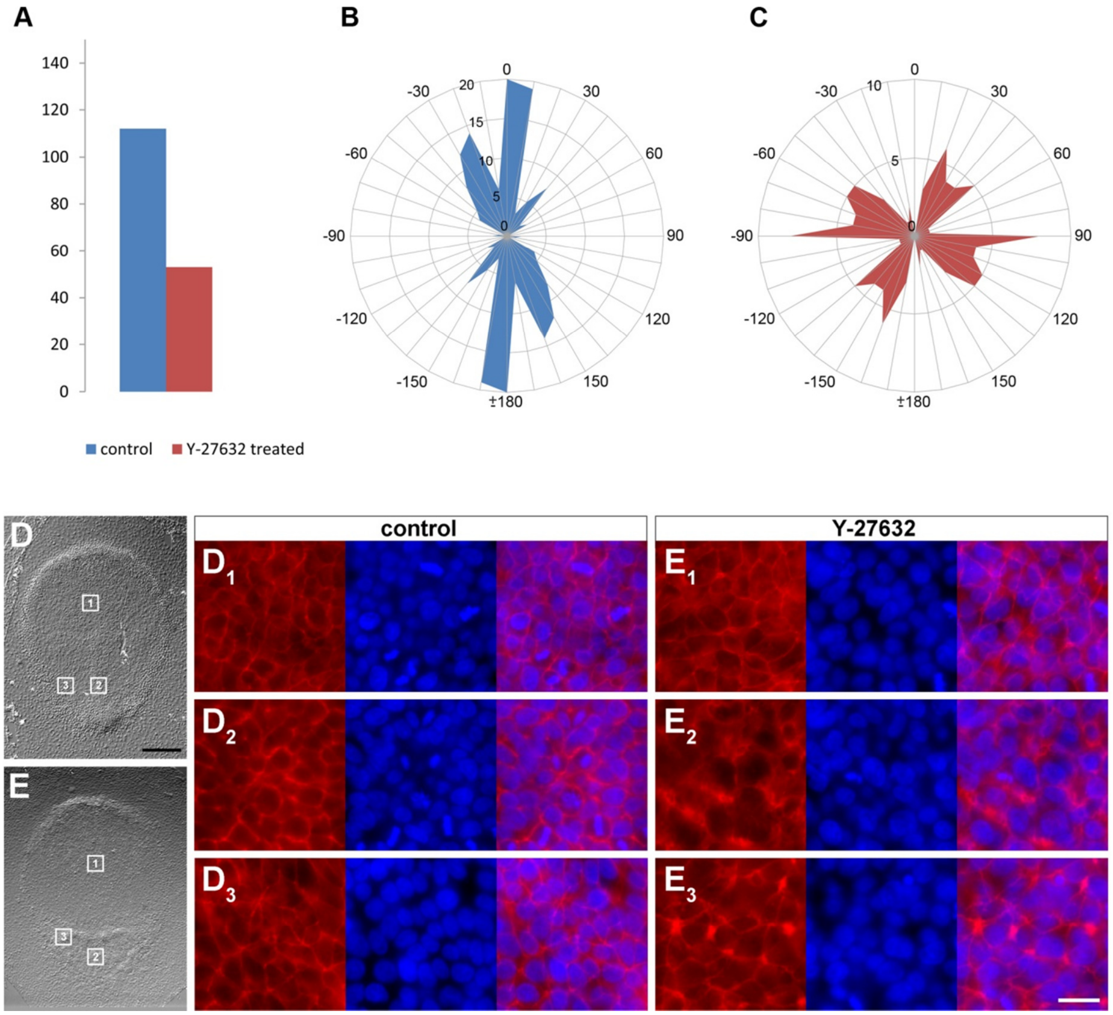

Fig. S1. Presumptive primitive streak area of ROCK-inhibited embryos reveals abnormality of orientation of cell divisions as well as of actin distribution. (A) Number of cell divisions used for the orientation of cell divisions counted in three control (*n*=112) and three embryos treated with Y-27632 (*n*=58) in the time period of 100 min. (B,C) Orientation of metaphase plates in relation to the AP axis (0°-±180°)

taken from the PGE area of control embryos (blue) and embryos treated with Y-27632 (red). Radial graduation shows the number of cells that fall within a specific angular region and orbital graduation shows the groups of angles (0°-±180°). (D,E) Dorsal views of control (D) and treated (E) embryo with high magnifications (D_1-E_3) of phalloidin-TRITC and DAPI staining in anterior (D_1, E_1) and posterior ($D_{2,3}$, $E_{2,3}$) regions (boxed in D and E). Clump-like actin distribution (foci) is found near the nucleus of epiblast cells in the presumptive primitive streak area of embryos treated with Y-27632, only. Scale bars: D, E, 250 µm; D_1-D_3, E_1-E_3 10 µm.

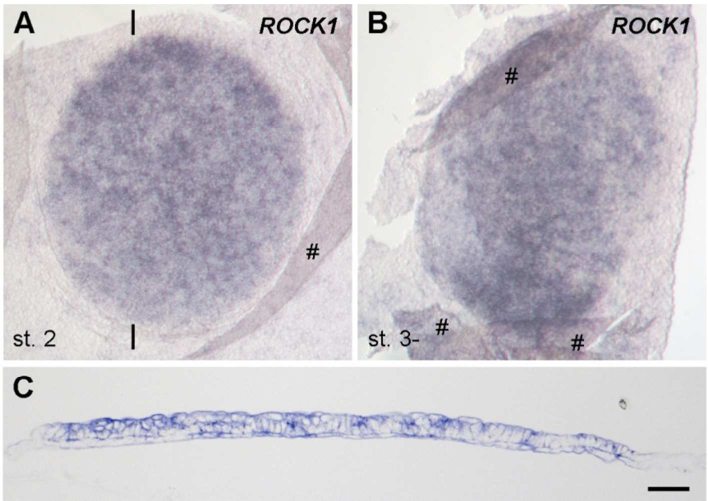

Fig. S2. Rho kinase (ROCK1) is expressed during the mammalian gastrulation. (A,B) Dorsal views of rabbit embryonic discs at stage 2, i.e. prior to primitive streak formation (A) and stage 3, i.e. during ongoing primitive streak formation (B). (C) Sagittal section from the embryo shown in A (note the black lines marking the level of the section). #, artefactual folds. Scale bars: A, B, 500 µm; C, 60 µm.

Supplementary Tables

Table S1. Dose-dependent primitive streak (PS) development

	Number of embryos treated with ROCK inhibitor (% total)			
	**control**<br>**(*n*=8)**	**10-30 µmol**<br>**(*n*=8)**	**40 µmol**<br>**(*n*=87)**	**50-100 µmol**<br>**(*n*=6)**
**Normal PS**	**8/8 (100)**	**0/8 (0)**	**6/87 (18)**	**0/6 (0)**
**Widened PS**	**0/8 (0)**	**8/8 (100)**	**50/87 (63)**	**4/6 (0)**
**No development**	**0/8 (0)**	**0/8 (0)**	**9/87 (19)**	**2/6 (33)**

Table S2. Forms of gastrulation centers and developmental stage at start of culture

	Number of embryos treated with 40 µmol ROCK inhibitor (% total)				
	**Stage 1** (*n*=4)	**Early stage 2** (*n*=17)	**Late stage 2** (*n*=44)	**Early stage 3** (*n*=14)	**Late stage 3** (*n*=8)
**Normal PS**	0/4 (0)	1/17 (6)	3/44 (7)	1/14 (7)	3/8 (38)
**WGC grade 1**	0/4 (0)	2/17 (12)	8/44 (18)	3/14 (21)	3/8 (38)
**WGC grade 2**	1/4 (25)	3/17 (18)	13/44 (30)	4/14 (29)	2/8 (25)
**WGC grade 3**	0/4 (0)	2/17 (12)	15/44 (34)	5/14 (36)	0/8 (0)
**No development**	3/4 (75)	9/17 (53)	5/44 (11)	1/14 (7)	0/8 (0)

PS, primitive streak; WGC, widened gastrulation center.

Supplementary Movie legends

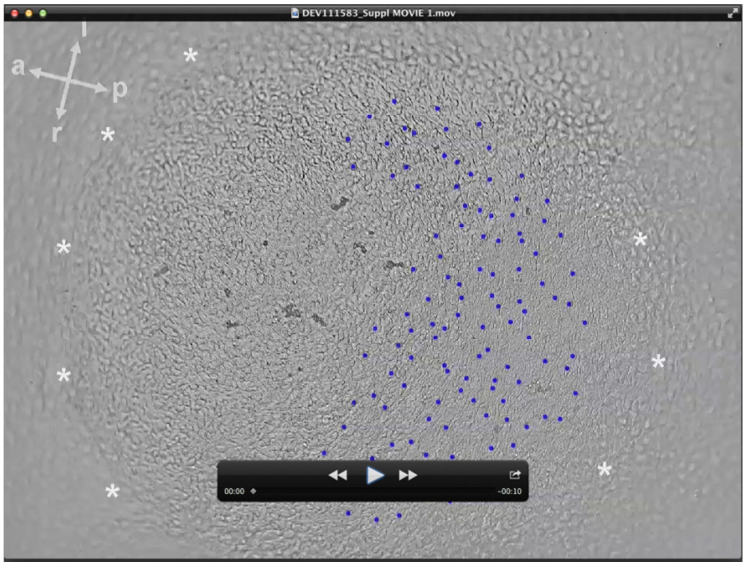

Movie S1. Gastrulation cell movements in control rabbit embryo. DIC time-lapse movie (first 190 min) of control embryonic disc shown in Fig. 1A-E (compare with box in A), starting immediately prior to gastrulation and using a 10x objective. Anterior is to the left. Asterisks mark the anterior and posterior borders of the embryonic disc. Blue tracks highlight individual cell movements in the posterior half of the embryonic disc. Note the primitive streak starting to form in the right half of the area shown. For details see legend of Fig. 1.

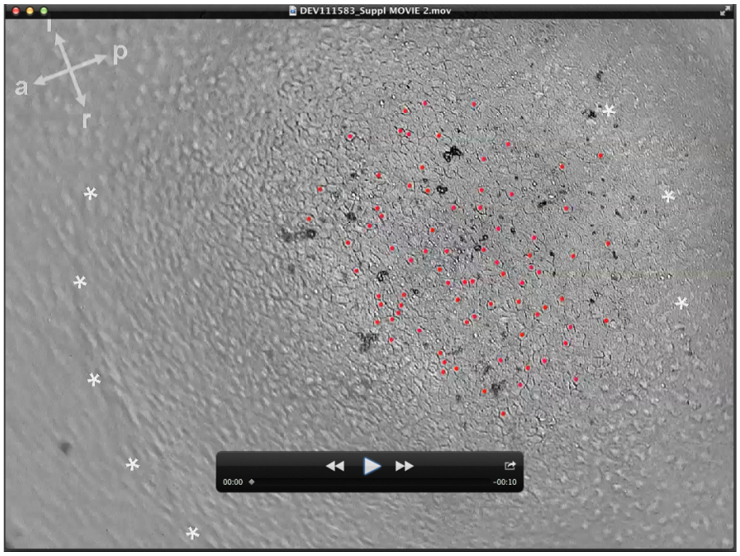

Movie S2. ROCK-inhibited gastrulation cell movements in the rabbit. DIC time-lapse movie (190 min) of ROCK-inhibited embryonic disc shown in Fig. 1F-J (compare with box in F), starting immediately prior to gastrulation and using a 10x objective. Anterior is to the left. Asterisks mark the anterior and posterior borders of the embryonic disc. Red tracks highlight centrifugal cell movements (instead of L- and U-turns) following straight paths towards the border of the embryonic disc. For details see legend of Fig. 1.

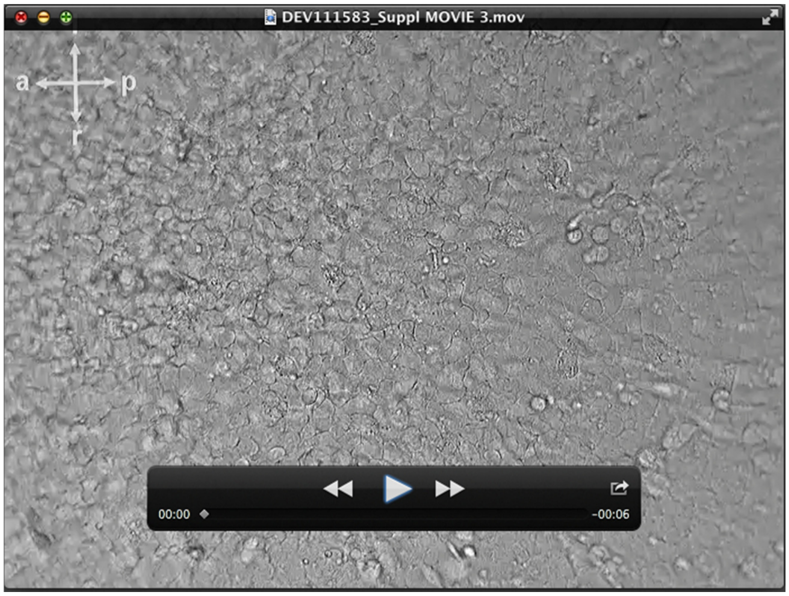

Movie S3. LatA-disturbed gastrulation cell movements in the rabbit. DIC time-lapse movie (120 min) of LatA-treated embryonic disc shown in Fig. 3F-H, starting immediately prior to gastrulation and using a 20x objective. Anterior is to the left. Epiblast cells present small oscillating movements, whereas hypoblast cells detach from the embryonic border, move towards the centre of the embryonic disc (note white arrows at the end of the movie) and build an artificial star-like structure in the centre of the embryonic disc. For details see legend of Fig. 3.
